# Ovarian Rhabdomyosarcoma in Children

**DOI:** 10.7759/cureus.85570

**Published:** 2025-06-08

**Authors:** Elily D Apumayta, Rolando Fernandez, Angela Chamochumbi, Edu Gomez, Vladimir Villoslada, Cecilia Ugaz

**Affiliations:** 1 Surgical Oncology, Instituto Nacional de Enfermedades Neoplasicas, Lima, PER; 2 Gynecologic Oncology, Instituto Nacional de Enfermedades Neoplasicas, Lima, PER; 3 Pathology, Instituto Nacional de Enfermedades Neoplasicas, Lima, PER; 4 Radiodiagnosis, Instituto Nacional de Enfermedades Neoplasicas, Lima, PER; 5 Pediatric Oncology, Instituto Nacional de Enfermedades Neoplasicas, Lima, PER

**Keywords:** advanced ovarian cancer, embryonal rhabdomyosarcoma (erms), metastatic rhabdomyosarcoma, ovarian neoplasms, rhabdomyosarcoma (rms)

## Abstract

Introduction: Rhabdomyosarcoma is the most common soft tissue sarcoma in children. It predominantly arises in parameningeal and periorbital regions, as well as in other areas that do not typically contain striated muscle. Ovarian involvement is rare and may be attributed to the presence of ovarian stromal fibroblasts, or endometriotic stroma. Ovaries may be considered a favorable site for rhabdomyosarcoma. The disease typically presents in children under three years of age, often at an advanced stage and with nonspecific symptoms. Management requires a multimodal treatment approach.

Methods: This was a retrospective analysis of ovarian rhabdomyosarcoma cases over the past 25 years at a national cancer referral center in Peru.

Results: Six female patients, aged between five months and 13 years, were included in the study. Four of them were classified as clinical stage and group IV due to the presence of distant metastases, while the remaining two were categorized as low-risk. The majority had embryonal histology, and none tested positive for fusion genes. All patients underwent chemotherapy and surgery. Surgical approaches varied according to disease extent, with an emphasis on fertility preservation. These ranged from unilateral adnexectomy with ovarian-sparing staging in cases of suspected localized disease, to primary cytoreduction without hysterectomy or contralateral adnexectomy in cases presenting with severe symptoms due to mass effect from advanced tumors. Chemotherapy regimens were selected based on risk stratification and aligned with international treatment protocols. Three patients received intensity-modulated abdominopelvic radiotherapy at a total dose of 2,400 cGy, delivered in 16 sessions for peritoneal sarcomatosis, two due to persistent disease following chemotherapy and surgery, and one due to high-risk histology. One low-risk patient achieved a survival of up to 94 months.

Conclusions: Ovarian rhabdomyosarcoma is rare. Its clinical and radiological manifestations are nonspecific. The use of immunohistochemistry is essential for diagnosis. Advanced disease with embryonal histology is predominantly observed. Multimodal treatment has achieved survival exceeding 87 months even in cases of metastatic stages.

## Introduction

Rhabdomyosarcoma is the most common soft tissue sarcoma in children. It arises from immature mesenchymal cells committed to the skeletal muscle lineage. However, primary tumors have been reported in anatomical sites lacking striated muscle. The disease predominantly affects the parameningeal and periorbital regions, and less frequently the trunk, pelvis, and retroperitoneum. Ovarian involvement is rare [1].

Ovarian malignancies are the most common gynecologic cancers in the pediatric population. In contrast to adults, where epithelial carcinomas predominate, germ cell tumors are more frequent in children. Overall, the risk of malignancy in adnexal masses among girls averages 19%, with reports ranging from 2% to 59% [2].

Therefore, the diagnosis of ovarian rhabdomyosarcoma (OR) requires the exclusion of more common primary ovarian neoplasms and metastatic disease. The occurrence of this rare entity may be attributed to the presence of ovarian stromal fibroblasts, or endometriotic stroma. The World Health Organization classifies rhabdomyosarcoma into four histological subtypes: embryonal, alveolar, pleomorphic, and spindle cell/sclerosing [3]. To date, approximately 50 cases of ovarian rhabdomyosarcoma have been reported worldwide [4-6].

OR is an aggressive malignancy that can occur at any age. Reported cases range from 13 months to 83 years of age, with no clear peak incidence during childhood. It is most commonly diagnosed at advanced stages (Stage III or IV) according to the Intergroup Rhabdomyosarcoma Study Group (IRSG) staging system. Although its rarity hinders a precise understanding of its natural history, the necessity of a multimodal treatment approach is well established. Abdominal radiotherapy may offer benefits in select cases. Surgery alone is insufficient. Chemotherapy has demonstrated superior short-term response rates; however, fewer than 50% of patients survive beyond three years [4,7,8].

This study details the clinical manifestations, management, and prognosis of pediatric patients diagnosed with OR over the past 25 years at the Instituto Nacional de Enfermedades Neoplasicas (INEN) in Lima, Peru. Staging and risk stratification were performed according to the IRSG criteria.

## Materials and methods

This was a retrospective review of the medical records of patients with ovarian rhabdomyosarcoma from INEN over the past 25 years. The results of clinical manifestations, diagnostic evaluations, treatment, and follow-up were registered. Uncertain diagnoses were excluded. Authorization was obtained from the local Institutional Review Board to review the medical records and publication of results.

## Results

Case one

**Figure 1:**
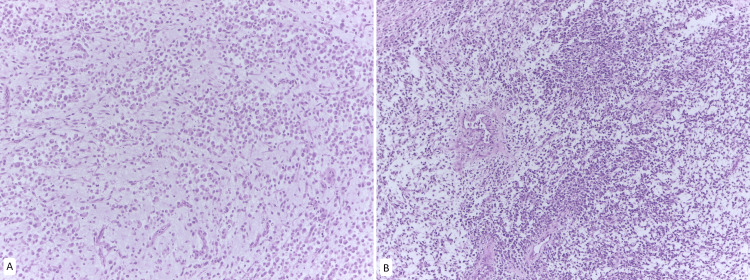
Parietal peritoneum metastases A. H&E stain at 10x magnification. Myxoid-appearing stroma with sarcomatous features B. H&E stain at 10x magnification. Presence of round cells with extensive eosinophilic cytoplasm

A five-month-old patient presented with progressive abdominal distension for one month, caused by a mixed, predominantly cystic tumor measuring 20 cm, originating from the left ovary. She underwent unilateral oophorectomy with conservative surgical staging, which revealed a primary embryonal rhabdomyosarcoma of the ovary associated with a teratoma, with predominance of the sarcomatous component (Figure 1). Metastases were identified in the omentum, the serosa of the small intestines, and the parietal peritoneum. The patient was classified as clinical group IV according to the IRSG system (T2bNxM1), indicating high-risk disease.

Then, the patient received chemotherapy with a local protocol consisting of five cycles of vincristine, doxorubicin, and cyclophosphamide, achieving an apparent complete radiologic response. However, three months after completing chemotherapy, a 15 cm heterogeneous, vascularized mass was detected, extending anterior to the ipsilateral kidney and encompassing the pancreas. A subsequent chemotherapy regimen with vincristine, doxorubicin, and platinum was administered, but the patient showed clinical progression. She was transitioned to palliative care and discontinued treatment one month later.

Case two

An 11-year-old patient presented with abdominal pain and nausea for the past two months, associated with a solid-cystic pelvic tumor. No distant metastases were identified. She underwent right oophorectomy due to the presence of a tumor confined to the right ovary, with no signs of carcinomatosis, in a non-oncology institution. The initial pathology report identified a high-grade spindle cell sarcoma, measuring 10 cm, limited to the ovary. She was subsequently treated with one cycle of chemotherapy for non-rhabdomyosarcoma tumors, based on ifosfamide and doxorubicin.

**Figure 2:**
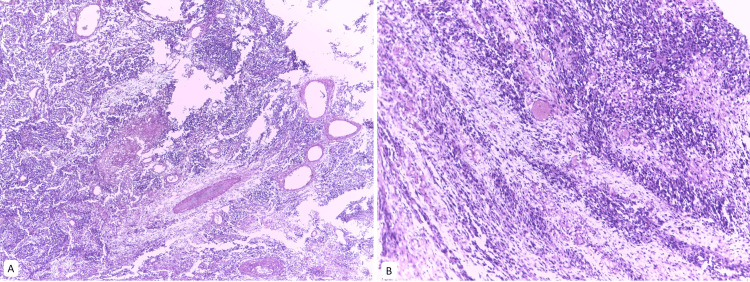
Embryonal rhabdomyosarcoma of the ovary Typical pattern often containing both hypocellular and hypercellular areas. A. H&E stain at 4x magnification. B. H&E stain at 4x magnification.

Following confirmation of the predominant sarcomatous component of embryonal rhabdomyosarcoma (Figure 2), associated with a mature teratoma of the ovary, the chemotherapy regimen was modified to the Children's Oncology Group (COG) ARST0331 protocol, which includes vincristine, actinomycin, and cyclophosphamide. According to the IRSG system, the tumor was classified as clinical group I (T1bN0M0), low-risk subgroup A. Evaluation at week 10 confirmed the absence of residual disease. Radiotherapy was not required. The patient remains event-free for six years.

Case three

**Figure 3:**
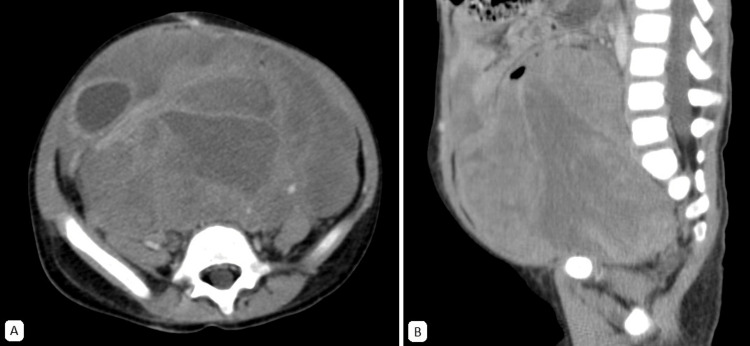
Abdominal CT scan, venous-phase. An extensive, predominantly solid, lobulated mass with heterogeneous contrast enhancement is observed. Hypodense areas, likely indicative of a cystic and/or necrotic component, measure approximately 10 x 14 x 12 cm. The epicenter is located in the pelvic cavity, extending into the abdominal cavity and exerting mass effect on peritoneal structures. A. Axial plane B. Sagittal plane

A two-year-old patient with a history of neurofibromatosis type 1 (NF1), NF1 microdeletion and variants of uncertain significance in the CASR, NF2, and POLD1 genes, presented with intestinal subocclusion caused by a 15 cm heterogeneous pelvic tumor (Figure 3).

**Figure 4:**
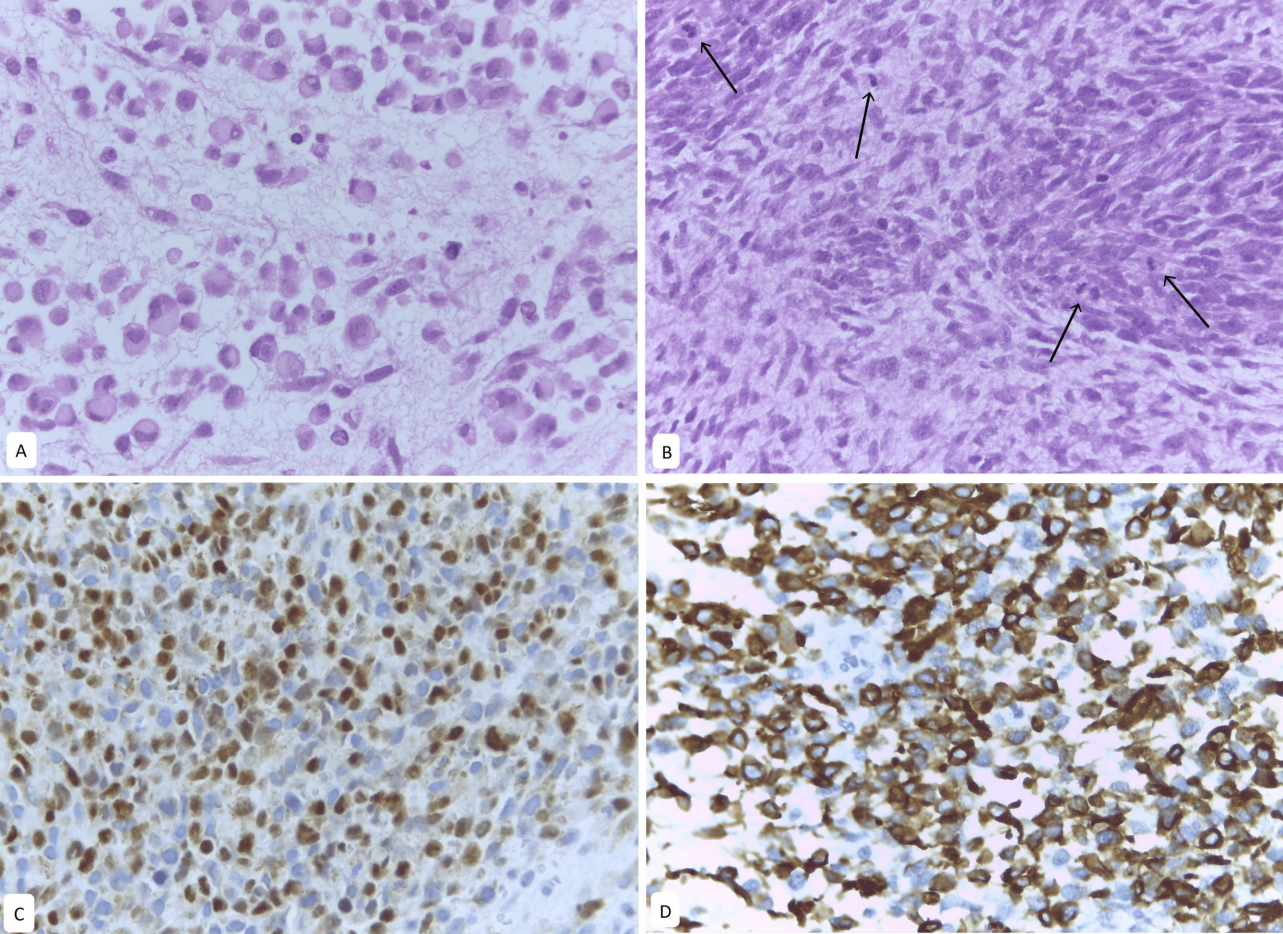
Metastatic embryonal ovarian rhabdomyosarcoma A. Serosal implant on the small intestine. Round tumor cells with wider eosinophilic cytoplasm. B. Left adnexal tumor. Presence of spindle and round cells with little cytoplasm with slight nuclear molding and presence of abundant mitotic figures (black arrows) C. Myogenin nuclear staining in tumor cells. D. Nuclear and cytoplasmic desmin positivity in tumor cells.

The patient underwent exploratory laparotomy, which revealed that the left ovary was replaced by a tumor with a ruptured capsule and irregular borders. Multiple diffuse implants, smaller than 5 mm, were observed on the ileal serosa, along with a 1 cm mesenteric lymphadenopathy. Suboptimal cytoreduction was performed, including unilateral oophorectomy. Initial frozen section analysis indicated a poorly differentiated malignant neoplasm, which was later confirmed as embryonal rhabdomyosarcoma (Figure 4). The tumor was classified as clinical group IV according to the IRSG system (T2bN1M1), indicating high-risk disease.

The patient then received chemotherapy according to the COG ARST0531 protocol, consisting of vincristine, doxorubicin, and cyclophosphamide. The chemotherapy course was irregular due to multiple infectious episodes. Total abdominal radiotherapy to the residual pelvic tumor, delivering 2400 cGy in 16 sessions and boost, with liver and kidney protection, reaching a total dose of 45 Gy using the volumetric modulated arc therapy (VMAT) technique. The patient remains event-free for six years.

Case four

**Figure 5:**
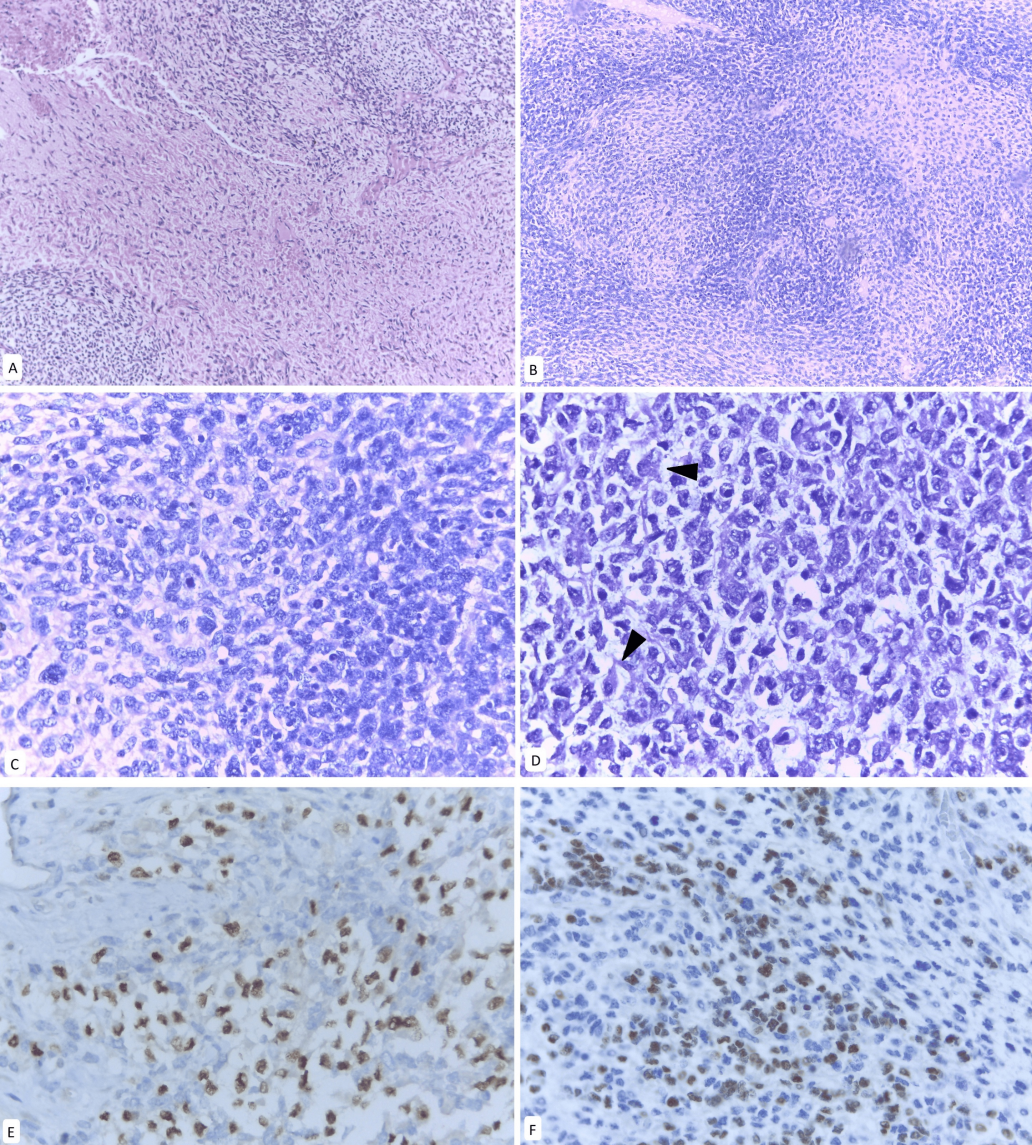
Ovarian embryonal rhabdomyosarcoma A and B. H&E stain at 10x magnification. Left ovary. Hypercellular areas of round cells and other more spindle-shaped cells with little cytoplasm and more eosinophilic hypocellular areas. C. Sheet of small cells, some stellate, round, and others spindle-shaped with little cytoplasm, occasionally others with a greater amount of eosinophilic cytoplasm. D. Cells with elongated tail-shaped cytoplasm (tadpole cells: black arrowhead). E. Myogenin nuclear staining in tumor cells. F. MYO D1 nuclear staining in tumor cells.

A 13-year-old patient with a history of a follicular thyroid tumor of uncertain malignant potential, previously treated with total thyroidectomy, presented with an incidental 7.5 cm pelvic mass. She subsequently underwent a left oophorectomy and conservative surgical staging. An ovarian embryonal rhabdomyosarcoma with anaplasia and a focus of hyaline cartilage was found (Figure 5). All biopsies obtained during staging were negative for malignancy. The tumor was classified as Group I according to the IRSG system (T1bN0M0), indicating a low-risk profile.

The disease progressed during chemotherapy, resulting in peritoneal carcinomatosis and implants on the liver capsule. The patient completed 25 weeks of high-risk chemotherapy following the European Pediatric Soft Tissue Sarcoma Study Group (European EpSSG) protocol, receiving ifosfamide, vincristine, actinomycin D, and doxorubicin (IVADO). She underwent an end-of-therapy mass excision consisting of an optimal secondary cytoreduction. Two well-defined, necrotic-appearing nodules, each measuring up to 1.5 cm, were identified in the hepatoduodenal and falciform ligaments. Histopathological analysis of these nodules was negative for malignancy. She subsequently completed maintenance chemotherapy with vinorelbine and cyclophosphamide. The patient remained event-free for two years.

Case five

**Figure 6:**
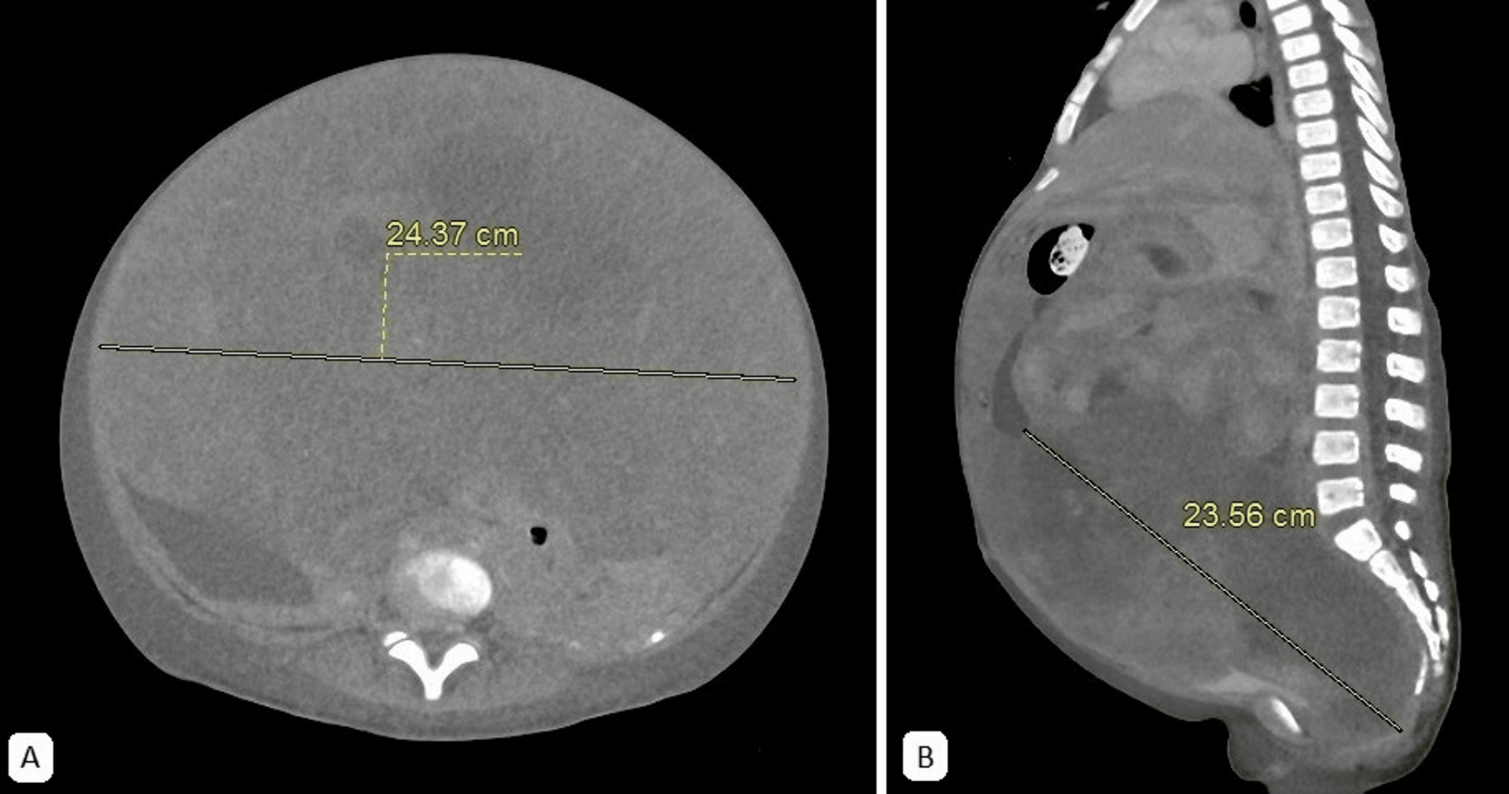
Abdominal CT scan, venous-phase. A. Axial plane. An extensive, predominantly solid mass with heterogeneous contrast enhancement is centered in the pelvic cavity and extends into the abdominal cavity. It is in close contact with the abdominal wall. B. Sagittal plane. There is diffuse thickening of the parietal peritoneum and irregular contours of the small intestine, associated with moderate free fluid in the abdominal cavity, consistent with peritoneal extension.

An eight-year-old patient presented with a one-month history of progressive abdominal distension, leading to severe pain and respiratory distress (Figure 6). Due to the symptomatic burden, an exploratory laparotomy and suboptimal primary ovarian cytoreduction were performed. Extensive peritoneal sarcomatosis and retroperitoneal masses were found. Histopathological analysis confirmed embrionary OR, with a tumor size of 38 cm. The disease was classified as Group IV according to the IRSG system (T2bN1M1), indicating a high-risk tumor

**Figure 7:**
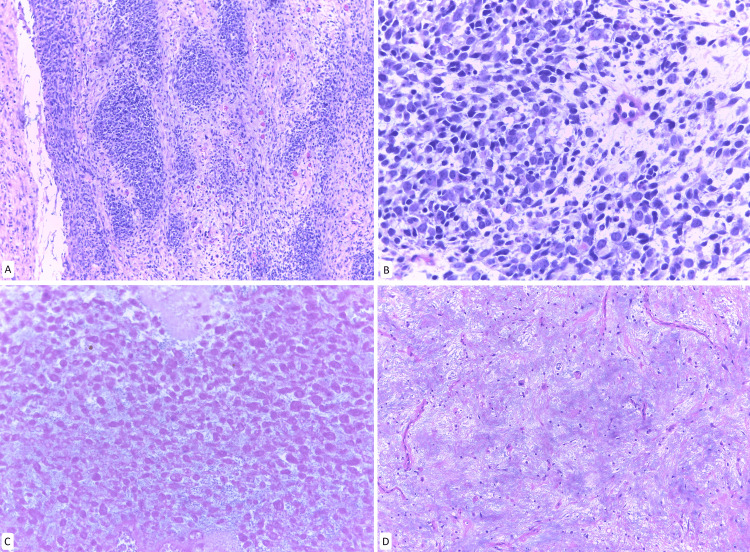
Viable post-chemotherapy rhabdomyosarcoma A. H&E stain at 10x magnification. Viable tumor cells, hypercellular but basophilic areas among hypocellular areas in eosinophilic stroma B. H&E stain at 40x magnification. Viable tumor cells, round with nuclear molding, some with scant cytoplasm and others with more eosinophilic cytoplasm. Presence of inconspicuous nucleoli. C and D. H&E stain at 10x and 40x magnification, respectively. Post-chemotherapy changes: Tumor necrosis and extensive areas of myxoid component.

The patient initiated chemotherapy with the EpSSG protocol. Due to clinical deterioration after the first cycle, subsequent treatment was discontinued until recovery and restarted at 75% of the planned dose. Re-evaluation at week 12 revealed persistent sarcomatosis and bulky retroperitoneal, perigastric, and perisplenic masses. She underwent a second suboptimal cytoreduction as an end-of-therapy surgery for OR. Pathology is shown in Figure 7.

She also received total abdominal radiation at a dose of 2400 cGy, delivered in 16 sessions using a three-dimensional (3D) conformal technique. Three months after completing maintenance chemotherapy with vinorelbine and cyclophosphamide, the disease progressed to involve the liver and spleen. She received one month of metronomic chemotherapy but ultimately succumbed to the disease.

Case six

**Figure 8:**
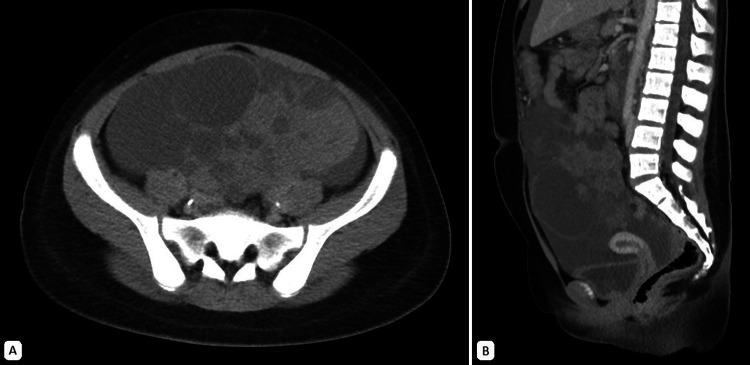
Abdominal CT scan, venous-phase. An extensive heterogeneous mass with lobulated borders and heterogeneous enhancement, likely of left adnexal origin, measuring 12.9 x 12 x 13.5 cm. It is associated with abundant free fluid in the abdominopelvic cavity, along with reticulations of the peritoneal fat planes. A. Axial plane B. Sagittal plane

A 10-year-old patient presented with a one-month history of abdominal pain and progressive abdominal distension. Imaging revealed an extensive mixed tumor measuring 17 × 14 cm originating from the right ovary, accompanied by abundant free fluid, mesenteric fat stranding, and a retroperitoneal mass measuring up to 7 cm (Figure 8). A 1.5 cm mediastinal lymphadenopathy was also noted on chest imaging. The initial serum cancer antigen 125 (CA-125) level was elevated at 453 U/mL (normal value < 35 U/mL).

**Figure 9:**
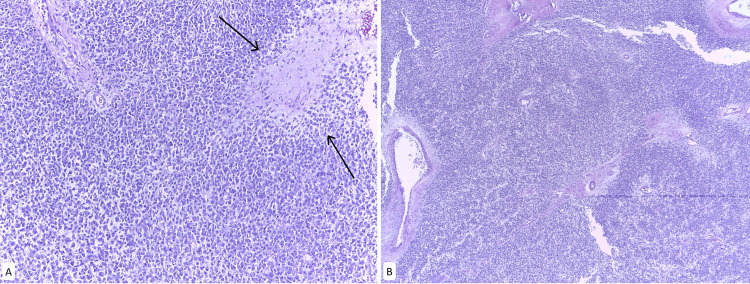
Alveolar rhabdomyosarcoma of the ovary A. H&E stain at 10x magnification. Loose myxoid stroma between tumor cells (black arrows) B. H&E stain at 4x magnification. Sheets of small, stellate, spiny, or round cells with scant cytoplasm

A percutaneous biopsy of the abdominal mass identified a poorly differentiated round cell malignancy, Desmin-positive, consistent with alveolar rhabdomyosarcoma (Figure 9). The patient started the high-risk EpSSG chemotherapy regimen. After 12 weeks, imaging demonstrated a partial response, with residual disease confined to the abdominal cavity. She subsequently underwent delayed primary excision via laparotomy, achieving optimal ovarian cytoreduction. The surgery included left adnexectomy, infracolic omentectomy, selective retroperitoneal lymphadenectomy, and wedge biopsy of the contralateral ovary. Histopathological examination revealed no evidence of a viable tumor.

The disease was classified as Group IV according to the IRSG system (T2bN1M1), indicating a high-risk tumor. Maintenance chemotherapy was continued with ifosfamide, vincristine, and actinomycin D (IVA); however, due to ifosfamide-associated seizures, the regimen was switched to vincristine, actinomycin D, and cyclophosphamide (VAC). Additionally, she received total abdominal radiation therapy at a dose of 2400 cGy in 16 sessions using the VMAT technique. The patient remained event-free for six months.

## Discussion

We present a case series of six girls, ranging from five months to 13 years of age, diagnosed with abdominal masses, all with pathological and immunohistochemical confirmation of OR, predominantly of the embryonal subtype. Each patient underwent comprehensive staging, including chest and abdominal CT scan and bone marrow biopsy. Rhabdomyosarcoma fusion genes were analyzed by real-time polymerase chain reaction, with no PAX-FOXO1 mutations detected in the alveolar case. All patients exhibited age-appropriate growth and development. To date, approximately 50 cases of ovarian rhabdomyosarcoma have been reported worldwide, excluding those cases with no consistent information about the rhabdomyoblastoma component. 

Even though INEN is a national referral cancer center, the small patient sample and the study’s restriction to a single institution are recognized as limitations. To address this, a summary of previously reported cases of patients with OR has been included (Table 1). This approach allows for a more comprehensive and representative description of clinical presentation, treatment strategies, and survival outcomes for OR. The first case series documented in the literature was conducted by Virchow in 1850 and included five patients. However, little information is available today [9-11] so they were not included. There are two other cases published in 1939 and 1929 by Lanza and Kuhn, respectively, mentioned by Sandison [11], but limited data is accessible. Cases describing rhabdomyoblastic components without a clear diagnosis of rhabdomyosarcoma were also not included.

**Table 1:** Patients diagnosed with ovarian rhabdomyosarcoma (OR), as reported in the literature NS, not stated; DOD, died of disease; DOS, died of sepsis; NED, no evidence of disease; PD: progression of disease; LF, lost to follow-up; UO, unilateral oophorectomy; USO, unilateral salpingo-oophorectomy; BSO, bilateral salpingo-oophorectomy; TH, total hysterectomy; SS, surgical staging; RT, radiotherapy; SLCT, Sertoli-Leydig cell tumor; CNS, central nervous system; ALL, acute lymphoblastic leukemia; GCT, germ cell tumor; VAC, vincristine, actinomycin D and cyclophosphamide; VDC, vincristine, doxorubicin and cyclophosphamide

N	Authors, et al	Year	Age, years	Clinical stage	Size, cm	Histology, associations and type	Treatment	Follow-up, months
1	Vignard [9]	1889	17	FIGO Ic	5	Pure. Pleomorphic type	NS	NS
2	Himwich [9]	1920	21	FIGO Ia	NS	Pure. Pleomorphic type	UO	DOD, 3.5
3	Barris [10]	1928	32	FIGO Ia	22	Pure. Pleomorphic type	UO. Abdominopelvic RT, dose NS	NS
4	Leopold [9]	1928	47	FIGO IIb	NS	Pure. Pleomorphic type	NS	NS
5	La Manna [9]	1936	57	FIGO III	NS	Pure. Pleomorphic type	NS	DOD, NS
6	Sandison [11]	1955	61	TxNxM0	NS	Pure. Embryonal type	BSO and TH	NED, 24
7	Rio [9]	1956	45	FIGO IIa	14	Pure. Pleomorphic type	BSO and TH. Pelvic RT	DOD, 15
8	Payan [9]	1965	86	FIGO IV	5	Pure. Embryonal type	None	DOD, 12
9	Srinivasa [9]	1967	35	FIGO III	30	Pure. Pleomorphic type	USO	LF
10	Dubey [9]	1967	40	FIGO IIb	18	Pure. Pleomorphic type	BSO and TH	NS
11	Spies [9]	1973	13 months	FIGO IV	22	Pure. Pleomorphic type	Ifosfamide	DOD, 18 days
12	Guérard [12]	1982	16	TxNxMx	20	Associated with SLCT. Pleomorphic type	USO. Cytoreduction of progressive peritoneal disease 6 months later. VAC	PD, 10
13	Guérard [9]	1983	32	FIGO Ia	10	Pure. Pleomorphic type	BSO and TH	DOD, 3
14	Nuñez [13]	1983	16	T2bN1M1	7	Pure. Embryonal type	Vincristine, Daunomycin, L-asparaginase, intrathecal Methotrexate. RT to CNS (as ALL)	DOS, 7
15	Zaloudek [14]	1984	16	FIGO Ia	15	Associated with SLCT. Type NS	Surgery NS. Thiotepa and 5-FU for recurrence 1,4 years later	DOD, 4
16	Nesland [15]	1985	78	TxNxM1	NS	NS	Chemotherapy NS	DOD, 12
17	Chan [16]	1989	4	TxNxM1	15	Pure. Embryonal type	BSO, TH and omentectomy. Chemotherapy NS	DOS, 7 days
18	Tsujimura [17]	1992	57	T1bNxM0	15	Associated with mucinous cystadenocarcinoma. Pleomorphic type	TH and BOS. Doxorubicin, Cisplatin, Cyclophosphamide	NED, 3
19	Amada [18]	1995	33	FIGO Ia	16	Associated with immature teratoma. Embryonal type	TH, BSO and appendectomy. VAC. Selective lymphadenectomy of recurrence. Metastasectomy of cervical lymph node. Cisplatinum, Vincristine, and Bleomycin	NED, 20
20	Nielsen [8]	1998	53	FIGO III	NS	Pure. Embryonal type	NS	DOD, 10
21	Nielsen [8]	1998	25	FIGO IV	NS	Pure. Embryonal type	NS	DOD, 7
22	Nielsen [8]	1998	22	FIGO III	NS	Pure. Alveolar type	NS	NED, 6
23	Nielsen [8]	1998	14	FIGO IV	NS	Pure. Alveolar type	NS	DOD, 26
24	Nielsen [8]	1998	10	NS	NS	Pure. Embryonal type	NS	LF
25	Nielsen [8]	1998	32	FIGO Ic	NS	Pure. Embryonal type	NS	LF
26	Nielsen [8]	1998	25	FIGO IIa	NS	Pure. Embryonal type	NS	DOD, 6
27	Nielsen [8]	1998	37	FIGO Ia	10	Pure. Embryonal type	TH, BSO, omentectomy, retroperitoneal lymphadenectomy. VDC	NED, 9
28	Nielsen [8]	1998	53	FIGO Ia	12	Pure. Embryonal type	TH, BOS and appendectomy. Doxorubicin, Ifosfamide, Vincristine	NED, 8
29	Nielsen [8]	1998	79	FIGO Ic	NS	Pure. Embryonal type	NS	NED, 2
30	Nielsen [8]	1998	69	FIGO IIa	NS	Pure. Embryonal type	NS	DOD, time NS
31	Nielsen [8]	1998	7	FIGO IIIc	NS	Pure. Embryonal type	NS	DOD, 17
32	Nielsen [8]	1998	62	FIGO III	NS	Pure. Embryonal type	NS	DOD, 10 days
33	Sant’Ambrogio [19]	2000	41	FIGO IIb	14	Associated with Clear cell carcinoma. Embryonal type	TH, BSO and SS. Doxorubicin, Ifosfomide, Carboplatin, Paclitaxel	DOD, 4
34	Sayhan [20]	2002	26	FIGO III	12	Pure. Alveolar type	HT and BSO, omentectomía. VAC	DOS, 5
35	Mikami [21]	2004	44	TxNxM1	8	Associated with adenosarcoma. Type NS	UO. Complementary cytoreduction with TH, omentectomy, contralateral SO and parts of small bowel. Ifosfamide and Cisplatin	Recurrence, 8
36	Grove [22]	2006	29	FIGO Ic	18	Associated with SLCT. Embryonal type	Tumorectomy, peritoneal washing. Complementary ipsilateral SO and omentectomy, 5 weeks later	NED, 4 years
37	Allende [23]	2008	25	TxNxM0	25	Pure. Embryonal type	USO, peritoneal washing and partial omentectomy. VAC. RT NS	NED, 11
38	Cribbs [7]	2008	13	TxNxM1	NS	Pure. Alveolar type	VDC. UO with en-block resection of colonic splenic flexure and omentum. RT NS. Oral Etoposide	Alive, 9
39	Cribbs [7]	2008	6	TxNxM1	NS	Pure. Embryonal type	USO, omentectomy en-bloc and peritoneal washing. VDC, Ifosfamide and Etoposide	Alive, 8
40	Rekhi [24]	2009	17	NS	25	Associated with SLCT. Embryonal type	USO. Excision of omental recurrence, TH and contralateral SO 1 year later	Recurrence, 1 year
41	Kefeli [25]	2009	65	NS	15	Associated with mature teratoma and contralateral serous carcinoma. Embryonal type	BSO, omentectomy and appendectomy. Platinum-based chemotherapy	NED, 3
42	Qureshi, [26]	2011	21	T1bN0M0	20	Pure. Pleomorphic type	USO and omentectomy. Complementary TH and contralateral SO	NED, 6
43	Ezem, [27]	2011	12	NS	22	Pure. Alveolar type	BSO	DOD, 17 weeks
44	Sangwan [28]	2012	10	IRSG I	14	Pure. Type NS	USO and peritoneal washing, peritoneal and omental biopsies. VAC	NED, 16
45	Al-Jumaily [29]	2012	12	N0M0	NS	Associated with mixed GCT. Embryonal type	USO, partial omentectomy, iliac lymph node sampling and peritoneal washing. BEP and VAC. Resection of recurrence	NED, 32
46	Bacalbasa [30]	2014	58	FIGO IIIc	NS	Associated with carcinosarcoma. Type NS	TH, BSO and total omentectomy R1 cytoreduction. Chemotherapy NS. R2 cytoreduction. Chemotherapy NS.	DOD, 7 months
47	Sanz-Baro [31]	2014	27	N0M1	18	Associated with mature teratoma. Pleomorphic type	USO. Complementary retroperitoneal lymphadenectomy, peritoneal washing, TH, contralateral SO and omentectomy. VAC	DOD, 4
48	Thway [32]	2014	60	TxN1Mx	11	Associated with carcinosarcoma. Pleomorphic type	Carboplatin and Paclitaxel. TH, BSO, retroperitoneal lymphadenectomy, omentectomy and appendectomy. Carboplatin and Paclitaxel.	Residual disease, 3
49	Tulek [33]	2014	64	FIGO IIIb	11	Associated with mature teratoma and chondrosarcoma. Type NS	TH, BSO, retroperitoneal lymphadenectomy and total omentectomy. Adriamycin and Ifosfamide. Resection of recurrent pelvic mass. Vincristine and Cyclophosphamide. Resection of second recurrent pelvic mass	DOD, 19
50	De Kock [34]	2015	6	NS	11,5	Pure. Embryonal type	USO and omentectomy. VAC, Etoposide, Ifosfamide. Total abdominal 18Gy RT	NED, 10 years
51	Chougule, [35]	2016	23	NS	25	Associated with SLCT and Borderline mucinous neoplasm. Spindle cell type	USO	NS
52	Benna [36]	2017	39	IRSG IIA	15	Pure. Pleomorphic type *Hermaphrodite male patient	Doxorubicine Dacarbazine and Ifosfamide. TH, BSO and SS. RT 45Gy and BOOST 15Gy	NED, 6
53	Vanidassane [4]	2018	21	IRSG IV	26	Pure. Embryonal type	USO, omentectomy and peritoneal washing. VAC	Alive, NS
54	Gökçe [37]	2020	12	IRSG IV	3	Pure. Alveolar type	Bilateral ovarian byopsy and omentectomy. Chemotherapy NS	Alive, NS
55	McCluggage [38]	2020	60	TxNxM1	28	Pure. Embryonal type	TH, BSO, infracolic omentectomy, partial pelvic peritonectomy, and appendectomy. Ifosfamide, vincristine, and actinomycin D. RT NS	Alive, NS
56	Oualla [39]	2020	19	TxNxM1	NS	NS. Alveolar type	VAC. Second line therapy with Etoposide and Ifosfamide	POD, NS
57	Srivastava [5]	2021	16	T1bNxM1	16	Pure. Alveolar type	USO, omentectomy, peritoneal biopsy and appendectomy. Chemotherapy NS	Alive, NS
58	Moinon [40]	2022	45	TxN1M1	18	Pure. Type NS	VAC	DOD, NS
59	Lethongsavarn [41]	2023	14	TxNxM0	10	Associated with teratoid, blastematous and neuroectodermal components. Embryonal type	UO, partial omentectomy, peritoneal biopsy. Ifosfamide, Vincristine and actinomycin-D	NED, 12 m
60	Bai [42]	2025	21	FIGO IV	21	Pure. Embryonal type	TH, BSO, pelvic lymphadenectomy, omentectomy and appendectomy. Chemotherapy NS	NED, 36
61	Bai [42]	2025	33	FIGO III	10	Associated with embryonal carcinoma. Embryonal type	Cytoreduction with TH, BSO, pelvic lymphadenectomy, omentectomy. Chemotherapy NS	DOD, 17
62	Bai [42]	2025	67	FIGO IIa	15	Associated with high grade serous adenocarcinoma. Embryonal type	TH and BSO. Chemotherapy NS	NED, 38

Most ovarian tumors are benign. Approximately 3% to 8% are malignant neoplasms, with the majority being germ cell tumors. A smaller proportion consists of sex cord, stromal and epithelial tumors. Imaging features such as solid consistency, size ≥8-10 cm, heterogeneous composition, and the presence of papillary projections raise suspicion for malignancy. These characteristics are often associated with elevated tumor markers, including human chorionic gonadotropin (HCG), alpha-fetoprotein (AFP), lactate dehydrogenase (LDH), CA-125, and inhibin [43]. In pediatric populations, reported series of ovarian tumors describe, in descending order of frequency, endometriomas, dermoid cysts, simple cysts, hemorrhagic cysts, dysgerminomas, mucinous or serous cystadenomas, and immature teratomas, among others [44]. Pediatric OR has been reported only in isolated case series (Table 2).

**Table 2:** Clinical manifestations, TNM, clinical stage histology, treatment and follow-up of pediatric patients with ovarian rhabdomyosarcoma (OR) NS, not stated; DOD, died of disease; DOS, died of sepsis; NED, no evidence of disease; PD: progression of disease; LF, lost to follow-up; UO, unilateral oophorectomy; USO, unilateral salpingo-oophorectomy; BSO, bilateral salpingo-oophorectomy; TH, total hysterectomy; SS, surgical staging; RT, radiotherapy; SLCT, Sertoli-Leydig cell tumor; CNS, central nervous system; ALL, acute lymphoblastic leukemia; GCT, germ cell tumor; VAC, vincristine, actinomycin D and cyclophosphamide; VDC, vincristine, doxorubicin and cyclophosphamide

N	Author, year	Age	Clinical manifestations. Related disease	TNM. Site of metastasis	FIGO Clinical Stage or IRSG Clinical Group	Histology	Treatment	Follow-up. State, months
1	Vignard, 1889 [9]	17 y	NS	NS	FIGO Ic	Pure. Pleomorphic type	NS	NS
2	Spies and Lorenz, 1973 [9]	13 m	NS	NS	FIGO IV	Pure. Pleomorphic type	Ifosfamide	DOD, 18 days
3	Guérard et al, 1982 [12]	16 y	Few months, lower abdominal pain and distension	TxNxMx	NS	Associated with SLCT. Pleomorphic type	USO. Cytoreduction for progressive peritoneal disease 6 months later. VAC	PD, 10 m
4	Nuñez et al, 1983 [13]	16 y	7 months, compression fracture of L1 vertebra, pain in the sternum, legs, and abdomen	T2bN1M1. Bone marrow, pancreas, brain, spinal cord, meninges, inguinal lymph nodes	NS	Pure. Embryonal type	Vincristine, Daunomycin, L-asparaginase, intrathecal Methotrexate. RT to CNS (as ALL)	DOS, 7 m
5	Zaloudek and Norris, 1984 [14]	16 y	NS	NS	FIGO Ia	Associated with SLCT. Type NS	Surgery NS. Thiotepa and 5-FU for recurrence 1,4 years later	DOD, 4 months
6	Chan et al, 1989 [16]	4 y	2 months, progressive abdominal distension, fever, anemia	TxNxM1. peritoneum	NS	Pure. Embryonal type	BSO, TH and omentectomy. Chemotherapy NS	DOS, 7 d
7	Nielsen et al, 1998 [8]	14 y	Time NS, rectal bleeding and bone pain.	Bilateral. Bone marrow	FIGO IV	Pure. Alveolar type	NS	DOD, 26 month
8	Nielsen et al, 1998 [8]	10 y	NS	NS	NS	Pure. Embryonal type	NS	LF
9	Nielsen et al, 1998 [8]	7 y	Acute abdominal pain	NS	FIGO IIIc	Pure. Embryonal type	NS	DOD, 17 m
10	Cribbs et al, 2008 [7]	13 y	Time NS, dyspnea and emesis.	TxNxM1. Bone, serosa of colon, lungs, mediastinal lymph nodes	NS	Pure. Alveolar type	VDC. UO with en-block resection of colonic splenic flexure and omentum. RT NS. Oral Etoposide	Alive, 9 months
11	Cribbs et al, 2008 [7]	6 y	1 month, vague abdominal pain and distension, anorexia, nausea, weight loss and emesis	TxNxM1. Pleural and peritoneal	NS	Pure. Embryonal type	USO, omentectomy en-bloc and peritoneal washing. VDC, Ifosfamide and Etoposide	Alive, 8 months
12	Rekhi et al, 2009 [24]	17 y	NS, vague abdominal pain and distension	NS	NS	Associated with SLCT. Embryonal type	USO. Excision of omental recurrence, TH and contralateral SO 1 year later	LF
13	Ezem et al, 2011 [27]	12 y	3 months, progressive abdominal distension, fever, weight loss	NS. Bilateral	NS	Pure. Alveolar type	BSO	DOD, 17 weeks
14	Sangwan et al, 2012 [28]	10 y	2 months, gradually increasing abdominal mass	TxN0M0	IRSG I	Pure. Type NS	USO and peritoneal washing, peritoneal and omental biopsies. VAC	NED, 16 months
15	Al-Jumaily et al, 2012 [29]	12 y	2 months, lower abdominal pain and distension, palpable non-tender abdominal mass	TxN0M0	NS	Associated with mixed GCT. Embryonal type	USO, partial omentectomy, iliac lymph node sampling and peritoneal washing	NED, 32 months
16	De Kock et al, 2015 [34]	6 y	3 months, abdominal distension, rigidity, difficulty passing urine	NS	NS	Pure. Embryonal type	USO and omentectomy. VAC, Etoposide, Ifosfamide. Total abdominal 18Gy RT	NED, 10 years
17	Gökçe et al, 2020 [34]	12 y	5 days, mild dyspnea, abdominal swelling	TxN1M1. Peritoneum, liver	IRSG IV	Pure. Alveolar type	Bilateral ovarian byopsy and omentectomy. Chemotherapy NS	Alive, NS
18	Srivastava et al, 2021 [5]	16 y	Time NS, enlarging pelvic mass, abdominal distension and pain	T1bNxM1. Axillary lymph node	NS	Pure. Alveolar type	USO, omentectomy, peritoneal biopsy and appendectomy. Chemotherapy NS	Alive, NS
19	Lethongsavarn et al, 2023 [41]	10 y	4 d, abdominal pain, palpable pelvic mass. DICER1 mutation	TxNxM0	NS	Associated with teratoid, blastematous and neuroectodermal components. Embryonal type	UO, partial omentectomy, peritoneal biopsy. Ifosfamide, Vincristine and Actinomycin-D	NED, 12 m
20	Apumayta et al, 2025	5 m	1 month, progressive abdominal distension	T2bN0M1. Peritoneum	IRSG IV	Associated with teratoma. Embryonal type	UO and conservative SS. VDC	LF, 13 m
21	Apumayta et al, 2025	11 y	2 months, abdominal pain and nausea	T1bN0M0	IRSG I	Associated with teratoma. Embryonal type	UO. Ifosfamide and Doxorubicin (as for NRSTS) and VAC	NED, 94 m
22	Apumayta et al, 2025	2 y	Acute intestinal subocclusion. NF1	T2bN1M1. Peritoneum, mesenteric lymph nodes	IRSG IV	Pure. Embryonal type	Suboptimal cytoreduction. VDC. Total abdominal 24 Gy RT + BOOST up to 45Gy	NED, 87 m
23	Apumayta et al, 2025	13 y	Incidental finding of pelvic mass. Follicular thyroid tumor	T1bN0M0	IRSG I	Associated with hyaline cartilage and anaplasia. Embryonal type	UO and conservative SS. IVADO. Vinorelbine and Cyclophosphamide	NED, 38 m
24	Apumayta et al, 2025	8 y	1 month, progressive abdominal distension and dyspnea	T2bN1M1. Peritoneum, perigastric and perisplenic lymph nodes	IRSG IV	Pure. Embryonal type	Suboptimal cytoreduction. IVADO. Second suboptimal cytoreduction. Total abdominal 24Gy RT	DOD, 12 m
25	Apumayta et al, 2025	10 y	1 month, abdominal pain and distension	T2bN1M1. Peritoneum, mediastinal lymph node	IRSG IV	Pure. Alveolar type	IVADO. Optimal cytoreduction. Ifosfamide, Vincristine and Actinomicin-D switched to VAC. Total abdominal 24Gy RT	NED, 17 m

The ovary is an uncommon primary site for rhabdomyosarcoma. However, it may be considered a favorable location, as it falls within the genitourinary tract, excluding organs such as the bladder, prostate, kidneys, and ureters. Approximately 80% of rhabdomyosarcomas arising in the genitourinary system are of the embryonal subtype, consistent with the cases in our series. OR has most commonly been reported in association with mature or immature teratomas, clear cell carcinoma, adenosarcomas, and Sertoli-Leydig cell tumors [38]. Its occurrence as a pure rhabdomyosarcoma is even rarer [8]. Case One and Case Two had a minor teratoma component associated with the OR.

The main symptoms of pediatric OR are abdominal pain and distension. The history of symptoms is an acute onset, or around three months. There was a unique case of seven months' history with medullary compression that was treated as acute lymphoblastic leukemia (ALL) due to its bone marrow involvement. Also one asymptomatic case was an incidental finding during a routine exam. In cases of severe abdominal distension, children may present with dyspnea, which was the main indication of primary cytoreduction for one of our patients. 

Several cancer predisposition syndromes are associated with specific malignant ovarian neoplasms. For example, WAGR syndrome, Denys-Drash syndrome, and Frasier syndrome are linked to gonadoblastoma; Ollier-Maffucci syndrome is associated with juvenile granulosa cell tumor; rhabdoid tumor predisposition syndrome type 2 is linked to hypercalcemic small cell ovarian carcinoma; and DICER1 syndrome is associated with Sertoli-Leydig cell tumor [43]. Case Four has not undergone sequencing studies or analysis for major genetic rearrangements to rule out DICER1 syndrome. In addition to a personal history of a follicular thyroid tumor of uncertain malignant potential, her ovarian mass pathology revealed a focus of hyaline cartilage without atypia. The presence of embryonal rhabdomyosarcoma with cartilaginous differentiation is suggestive of DICER1 alterations [34,38,41]. This syndrome is also characterized by the presence of gynandroblastoma in the ovaries, cystic nephroma and Wilms tumor in the kidneys, as well as thyroid tumors exhibiting a follicular pattern [45].

NF1, commonly associated with neurofibromas, optic gliomas, and peripheral nerve sheath tumors, confers a 20-fold increased risk of developing rhabdomyosarcoma compared to the general population. This condition is primarily associated with the embryonal subtype of rhabdomyosarcoma, which typically occurs in the genitourinary system, with the bladder, prostate, and paratesticular regions being the most common sites, in order of frequency. The common age of presentation, as described in the literature, is less than three years, with a maximum age of 5.4 years. Rhabdomyosarcoma in patients with NF1 is often less metastatic than that in patients without genetic predisposition syndromes. Case Three, a two-year-old female with metastatic embryonal-type rhabdomyosarcoma, had genetic confirmation of NF1 [45,46]. Even though it was not a localized disease, she has a three-year overall survival. 

Histopathological evaluation of excised or biopsied material can be challenging in sarcomas due to the diversity of their characteristic histological presentations. According to the World Health Organization, there are currently four subtypes of rhabdomyosarcoma: embryonal, alveolar, pleomorphic, and spindle cell [3]. Embryonal rhabdomyosarcoma is the most common subtype and is characterized by aggregates of spindle and pleomorphic cells with hyperchromatic nuclei, rhabdomyoblasts, and myxoid stroma [3,24]. It consists of primitive mesenchymal cells that exhibit varying degrees of skeletal muscle differentiation. The tumor is moderately cellular, with a typical pattern featuring both hypercellular and hypocellular areas, along with a loose myxoid stroma. Perivascular condensations of tumor cells are also commonly observed in the less cellular regions [47]. The majority of children (17 of 25 cases) had a pure OR. The rest of OR were associated with another histologic component, such as Sertoli-Leydig cell tumor or teratoma.

Rhabdomyosarcoma is cytologically composed of small, stellate, spiny, or round cells with scant to deeply eosinophilic cytoplasm and small, eccentric, oval nuclei with inconspicuous nucleoli. Occasionally, tumor cells with increased eosinophilic cytoplasm can be identified, indicating characteristic rhabdomyoblastic differentiation. This differentiation may become more pronounced in patients with prior chemotherapy [3,5], as observed in Case Six. Rhabdomyosarcomas may also exhibit cells with elongated cytoplasmic tails, known as tadpole cells [3,6]. Immunohistochemical profiling is essential for the diagnosis of rhabdomyosarcoma. These tumors are positive for muscle differentiation markers such as desmin, and also for MyoD1 or myogenin. They are negative for pancytokeratin, ACL, inhibin, Melan-A, and S100, which helps exclude other origins, such as carcinoma, lymphoma, melanoma, or sex cord tumors - differential diagnoses of this pathology [47].

Ultrasonography (US) is often the first imaging modality used in the diagnosis of rhabdomyosarcoma. It is generally accessible, provides valuable information about the mass, and can assist in establishing a differential diagnosis. In the US, rhabdomyosarcoma typically appears as a well-defined, slightly hypoechoic, heterogeneous mass, which may demonstrate increased blood flow [48]. On CT scan, the tumor is characterized by a large, heterogeneous appearance with contrast enhancement (Figures 3, 6, 8). This modality is particularly useful for evaluating locoregional lymph nodes and detecting distant metastases, such as those in the lungs, liver, and bones [48].

When abdominal and pelvic sarcomas are suspected, magnetic resonance imaging (MRI) is the method of choice, as it provides a comprehensive view of the primary tumor, its locoregional extension, and the associated lymph nodes [49,50]. However, due to the heterogeneity of their morphology, as well as the presence of necrosis and hemorrhage, sarcomas do not exhibit a specific imaging pattern. Typically, they demonstrate intermediate signal intensity on T1-weighted sequences, and are either hyperintense or isointense on T2-weighted sequences, with prominent post-contrast enhancement. Functional diffusion-weighted imaging (DWI) offers valuable information on cell density, and diffusion restriction suggests a malignant etiology [48-50]. Due to limited access to MRI in our region, our patients underwent CT scans as the standard method for disease localization and staging.

Almost half of pediatric patients with OR had metastases at diagnosis, and only 20% had localized disease. This was contrary to what is set by the SEER database analysis, which found 49% of localized disease in patients with rhabdomyosarcoma, and 27% of metastatic disease [51]. The main site of metastasis for OR was the peritoneum, followed by distant lymph nodes and bone marrow. There are reported cases of pleural, lung and bone metastasis. The patient with leukemia-like presentation had also metastases in the inguinal lymph nodes, pancreas, brain, spinal cord, and meninges [13].

The stage of the disease guides treatment decisions. Treatment for advanced disease is multimodal, as seen in most of our patients. Surgery was a common element in all cases. Two patients (Case One and Case Four) underwent unilateral oophorectomy with ovarian-conserving staging. One patient, initially treated at a non-oncology institution (Case Two), received only unilateral oophorectomy. Two patients (Case Three and Case Five) underwent suboptimal cytoreduction due to severe symptoms resulting from the mass effect, which necessitated primary surgery. Another patient (Case Six) underwent biopsy to initiate chemotherapy, followed by optimal cytoreduction. Similarly, optimal cytoreduction was achieved after chemotherapy in Case Four, who experienced disease progression during adjuvant therapy. The first five cases underwent surgery without prior histological diagnosis, based on suspicion of ovarian malignancy guided by imaging and the exclusion of germ cell tumors (GCT), as tumor markers were negative, and epithelial neoplasia as a less probable differential diagnosis. 

In relation to the above mentioned, conservative surgical ovarian staging was performed due to suspected localized disease in the pediatric patient. This procedure involves unilateral salpingo-oophorectomy with preservation of the contralateral ovary and uterus, systematic inspection and palpation with peritoneal fluid cytology, multiple peritoneal biopsies, infracolic omentectomy, and lymph node assessment. This approach is also the standard surgical option for adolescent and young adult patients seeking fertility preservation in ovarian cancer with low malignant potential or low risk, such as clinical stage I epithelial tumors, borderline tumors, malignant stromal tumors, and sex cord tumors. It is preferred because it does not compromise event-free survival (EFS) or overall survival (OS) when compared to more radical surgical procedures. This conservative approach can be omitted in early-stage GCT or other ovarian tumors in the pediatric population [7,52,53].

The scientific evidence supporting fertility-sparing surgery (FSS) for ovarian rhabdomyosarcoma is limited due to its low incidence. The use of this approach was based on the finding of localized disease confined to one ovary in a pediatric patient. We believe that applying this criterion to OR is appropriate. In Table 2, the trend to perform FSS since the previous century is visible. There were only two exceptions (Chan et al. 1989 and Ezem et al. 2011, which described primary bilateral salpingo-oophorectomy (BSO) with total hysterectomy (TH) for a metastatic disease, and BSO for a bilateral disease. The clinical stage was not stated, then the survival cannot be representative. Even in cases with bilateral involvement, we do not recommend the primary BSO due to the age and high chemosensitivity of this diagnosis. Those are important differences with adults, who may be satisfied with their parity, and show less responsiveness to chemotherapy. As seen in Table 1, there are many cases treated with BSO and TH as part of the surgical staging (SS). 

Also in Table 2, there are patients who had conservative SS without lymph node assessment, such as lymphadenectomy or sampling. The role of lymph node assessment for OR in survival outcomes cannot be conclusive from these case reports, as has been clearly established in cases of paratesticular and extremity rhabdomyosarcoma. However, based on our experience in ovarian cancer, we believe in the necessity of lymph node sampling as a standard step in SS to confirm the stage and consequently to give the appropriate therapy. 

In patients who underwent cytoreduction, whether primary or interval, neither hysterectomy nor contralateral oophorectomy was performed. The procedure included oophorectomy of the involved ovary, infracolic omentectomy, and resection of the macroscopically visible tumor. Systematic lymphadenectomy was not performed, as it does not provide a survival benefit when clinically normal lymph nodes are present. Conversely, it is associated with greater postoperative morbidity [54]. In patient Case Six, due to the presence of retroperitoneal adenopathy, selective lymphadenectomy was performed. In this case, optimal cytoreduction was achieved after neoadjuvant therapy, defined as residual disease no greater than 1 cm in diameter. This differs from the two suboptimal primary cytoreductive surgeries (Case Three and Case Five), where residual disease was greater than 1 cm.

A core biopsy was performed in Case Six for histological diagnosis prior to the initiation of neoadjuvant therapy. The pretreatment biopsy should obtain sufficient tissue for immunohistochemical staining and additional genetic or molecular analysis. Neoadjuvant chemotherapy for rhabdomyosarcoma at other sites in the female reproductive tract, such as the vagina, cervix, and uterus, can achieve definitive local control, thereby avoiding surgery. In cases of incomplete response to chemotherapy, local radiotherapy and/or surgery may be required [55]. Our patient underwent delayed primary excision to address resectable residual disease after 12 weeks of chemotherapy. Biopsies for ovarian tumors are generally not recommended due to the risk of tumor spillage and upstage, except in cases of carcinomatosis, unresectable disease, or inoperable patients, where primary surgery would not be beneficial. In such cases, confirmatory pathology is necessary to initiate the appropriate chemotherapy regimen. Consequently, preoperative biopsies were not performed in Case Two and Case Four, as examples.

Among pediatric patients, 17 received primary surgery; meanwhile, chemotherapy was the most frequent treatment offered. The chemotherapy regimens for rhabdomyosarcoma used at our institution were initially based on a combination of vincristine, adriamycin, and cyclophosphamide. Currently, protocols are selected according to the risk group, which has facilitated the standardization of regimen selection. Accordingly, we use the COG ARST0331 regimen for low-risk disease, the COG ARST0531 regimen for intermediate-risk disease, and the European EpSSG regimen for high-risk disease.

Abdominal irradiation using VMAT is both effective and well-tolerated. Particularly in patients with peritoneal dissemination, a dose of 24 Gy in 16 fractions directed to the whole-abdominopelvic irradiation is suggested, a strategy supported by the Children’s Oncology Group to achieve local control of the disease [56]. The survival with that minimum dose of 24 Gy for metastatic abdominal rhabdomyosarcoma showed a three-year overall survival of 84% for those treated with radical irradiation, 54% for those with partial irradiation, and 23% for those who did not receive irradiation [57]. Three of our patients with clinical stage 4 disease due to peritoneal sarcomatosis received 2400 cGy in 16 sessions across a total abdominal field using either the VMAT or 3D technique. 

Nowadays, the use of VMAT and intensity modulated radiation therapy (IMRT) has reduced the toxicity associated with treatment compared to older techniques. These technologies have also made it possible to respect dose limits to critical organs, such as the liver and kidneys, thus improving the safety and effectiveness of the treatment [56]. Case Three and Case Five received radiotherapy due to persistent disease following chemotherapy and surgery. Case Six received radiotherapy for high-risk alveolar histology with advanced disease at presentation, specifically to reduce the risk of recurrence due to peritoneal sarcomatosis.

Pediatric patients with OR had a median age of 11 years (range: five months to 17 years), with 68% aged 10 years or older. The youngest patient was five months old, presented with high-risk rhabdomyosarcoma, classified as clinical group IV, and experienced clinical progression during chemotherapy. She survived for 13 months after diagnosis before abandoning follow-up. Being one year of age or younger has been described as a negative prognostic factor affecting event-free survival [58]. This may be related to the limitations of oncological treatments in this age group, which are constrained by the need to minimize toxicity and avoid interfering with proper developmental growth.

Factors associated with a lower likelihood of cancer-related death included white race, localized stage of disease, embryonal histologic type, and surgical treatment [51]. The pediatric patients who died of disease were predominantly reported before 2000, with clinical stage ≥ IIIc but no clearly stated treatment. We had an eight-year-old patient with a pure embryonary OR IRSG IV who died of disease 12 months after diagnosis in spite of multimodal treatment (Case Five). Case Three shows a similar scenario but with an opposite outcome. The differential factor among them was the general status of the patients and the consequent response to therapy. What forced us to interrupt and then decrease the dose of chemotherapy for Case Five.

Additionally, an important factor beyond histologic type, associated with a higher risk of therapeutic failure and death is the genetic alteration PAX-FOXO1 [59]. Of the six total pediatric alveolar OR, one was ours with no PAX-FOXO1 mutation. The rest five did not specify this factor, most of them due to a publication date previous to the widespread of this conduct. 

The survival rates reported in various pediatric case series show a maximum follow-up of 26 months (Table 2). One of our patients with low-risk localized T1b disease, clinical group I, was treated with R0 surgery followed by the adjuvant ARST0331 chemotherapy regimen, and achieved event-free survival for six years. In relation to this, IRSG I rhabdomyosarcoma of the female genitourinary tract has shown an OS of 88% at five years, with an EFS of 89% [58]. On the opposite side, a patient with clinical group IV disease, treated with suboptimal cytoreduction, the ARST0531 chemotherapy regimen, and radiotherapy due to persistent disease, achieved EFS for six years. This demonstrates the need for individualized decision making and multimodal therapy for OR.

## Conclusions

The ovary is a rare site for the development of primary rhabdomyosarcoma; however, based on most recent case reports with multidisciplinary approach, it may not be considered an unfavorable location. The clinical manifestations of ovarian rhabdomyosarcoma vary depending on the stage of the disease. In localized cases, it may be discovered incidentally or present with symptoms such as nausea or abdominal pain. In contrast, advanced tumors may manifest with progressive abdominal distension, leading to severe pain, respiratory distress, or even intestinal subocclusion. The predominant age group affected were pubescents and adolescents, compared to isolated cases in children. Radiological findings are typically nonspecific.

The diagnosis of ovarian rhabdomyosarcoma requires the exclusion of metastatic disease and other, more common primary ovarian neoplasms. Immunohistochemical and molecular analyses are essential for establishing an accurate diagnosis. The embryonal type is more frequently observed than the alveolar one. At the time of diagnosis, most patients are classified as clinical group IV, due to metastatic spread, most commonly involving the peritoneum. 

The complexity of ovarian rhabdomyosarcoma underscores the need for specialized multimodal treatment. Surgery alone is insufficient and should be tailored to preserve fertility whenever feasible. Chemotherapy is invariably required, with regimens adapted to the patient's individual risk category. Radiotherapy may contribute to local control in selected cases. Multimodal therapy has demonstrated effectiveness in achieving disease control, with reported survival rates exceeding six years, even in cases with metastatic disease.
